# Sudden cerebral depression detected by bispectral index monitoring in cryptococcal meningitis with elevated near‐fatal cerebrospinal fluid pressure

**DOI:** 10.1002/ams2.276

**Published:** 2017-05-28

**Authors:** Hironori Matsumoto, Suguru Annen, Kensuke Umakoshi, Jun Takeba, Satoshi Kikuchi, Yuki Nakabayashi, Naoki Moriyama, Muneaki Ohshita, Mayuki Aibiki

**Affiliations:** ^1^ Graduate School of Medicine Department of Emergency and Critical Care Medicine Ehime University Tohon City Ehime Japan

**Keywords:** bispectral index, cryptococcal meningitis, spinal drainage

## Abstract

**Case:**

An increase in cerebrospinal fluid pressure (CSFP) is usually prominent in cryptococcal meningitis, which has a high mortality rate, so aggressive management to control CSFP is crucial. In this case, a 40‐year‐old‐man survived cryptococcal meningitis treated with continuous spinal drainage under bispectral index (BIS) monitoring. He unexpectedly showed hypertension, went into a coma, and even loss his light reflexes due to CSFP elevation. His BIS values had abruptly dropped before developing these symptoms, but dramatically recovered after lumbar puncture drainage, suggesting that BIS monitoring could reflect cerebral function changes due to CSFP alternations.

**Outcome:**

Inducing continuous spinal drainage to control CSFP provided stable control of blood pressure and brain activity, which was continuously monitored by BIS, enabling us to provide prompt treatment.

**Conclusion:**

Cerebral depressions due to elevated CSFP may suddenly develop, so continuous spinal drainage is needed for preventing catastrophic events. Bispectral index could be useful for detecting early changes from CSFP elevation in meningitis cases with intracranial hypertension.

## Introduction

Cerebrospinal fluid pressure (CSFP) elevation is common in cases of cryptococcal meningitis and is associated with high mortality.^1^ We describe a case of cryptococcal meningitis with CSFP of >50 cmH_2_O, who showed sudden depressions in cerebral function. We could control CSFP with continuous spinal drainage and temperature management without complications. Furthermore, bispectral index (BIS) monitoring well reflected changes in cerebral activities and response to treatment.

## Case

A 40‐year‐old man was admitted to a regional hospital due to suspected meningitis. He presented with seizures and impaired consciousness, so was transferred to our university hospital.

On arriving, he had a disturbance of consciousness without fever. Although a brain computed tomography scan did not reveal remarkable changes, including hydrocephalus, the patient had high CSFP of >50 cmH_2_O. A microscopic cerebrospinal fluid (CSF) examination showed *Cryptococcus neoformans*. Amphotericin B 300 mg/day and flucytosine 5000 mg/day were given for fungal meningitis. Intermittent lumbar punctures were scheduled to control the raised CSFP.

After the admission the patient's consciousness gradually declined to the critical level so as to obstruct airways with high fever. Cerebrospinal fluid pressure still reached above 50 cmH_2_O and lumbar puncture drainage did not improve his consciousness, so we carried out respiratory management with tracheal intubation. We also considered encephalitis and brain swelling due to the progression of *C. neoformans*. The patient's body temperature was reduced to normothermia with a surface cooling device against his fever elevation, which might worsen cerebral injuries from infection, then started neurological monitoring with BIS on day 2. Several hours after inducing normothermia, his blood pressure suddenly increased (208/119 mmHg) and his light reflex disappeared, so we started a barbiturate (thiamylal 750 μg/kg/h) for the purpose of reducing CSFP, and decreased his temperature to 34°C with continuous administration of neuromuscular blockade (NMB). After the intervention, blood pressure became normal and stabilized, and light reflex was regained. A brain computed tomography scan taken at the time revealed no remarkable changes.

**Figure 1:**
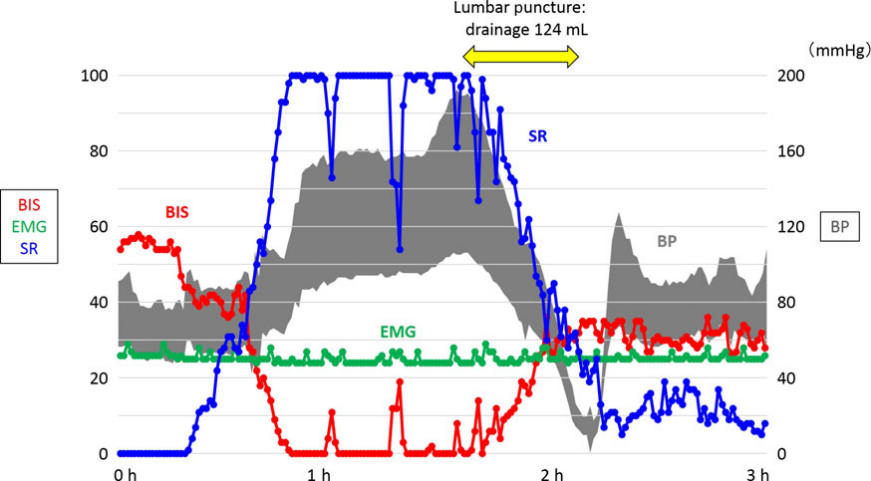
Clinical course of a 40‐year‐old‐man with cryptococcal meningitis and elevated near‐fatal cerebrospinal fluid pressure (CSFP), monitored by bispectral index (BIS). On day 2, soon after administration of neuromuscular blockade, BIS electromyography (EMG) values sharply decreased to approximately 30, which means “zero levels” of electromyography signals. BIS values suddenly decreased to zero, which occurred before blood pressure (BP) rose, suggesting that BIS can show an earlier change in brain activity in response to CSFP elevation. We carried out an urgent therapeutic lumbar puncture and drainage, but the opening pressure was still >50 cmH_2_O. During CSF drainage at the volume of 124 mL, BIS values gradually recovered. Due to possible response to rapid decrease in CSFP, BP dropped profoundly and we stopped the drainage. After regaining BP, we lowered the body temperature further to 33°C for the purpose of controlling CSFP. SR, suppression ratio.

However, several hours after inducing mild hypothermia of 34°C, BIS values suddenly decreased to zero, meaning an isoelectric electroencephalogram, which had occurred approximately 15 minutes before blood pressure elevated and light reflex was lost. We carried out an urgent lumbar puncture and repeated CSF drainage as the CSFP was still >50 cmH_2_O. After CSF drainage at a volume of 124 mL, BIS values gradually recovered. However, blood pressure profoundly dropped, which might have been due to a rapid decrease in CSFP, so that we stopped the drainage. After regaining blood pressure, we reduced the target temperature further to 33°C for the purpose of reducing CSFP (Fig. 1).

On day 3, we inserted a spinal drain tube for controlling CSFP and preventing the risk of brain herniation during intermittent lumbar punctures. While the continuous CSF drainage was set at 10 cm H_2_O, BIS values and blood pressures returned to normal range. After a very gradual rewarming at 1°C/day and discontinuing treatment with the barbiturate, the patient was stable and extubated on day 8. Magnetic resonance imaging of the brain on day 9 depicted T2 high‐intensities at the bilateral basal ganglia, which might be due to the cryptococcomas. However, with good response to treatment, we removed the spinal drainage tube on day 27 and 90 days after admission he could walk around and was clinically asymptomatic.

## Discussion

As shown in this report, CSFP elevation produced not only systematic responses such as blood pressure elevation and loss of light reflexes, but also cerebral depressions. Furthermore, cryptococcal meningitis drove an abrupt development of these responses due to markedly elevated CSFP. The increase in CSFP is common in cryptococcal meningitis. Although still poorly understood, the cause of the raised CSFP is thought to be outflow obstruction by mechanical blockage of the passage of CSF across arachnoid villi. Furthermore, our patient had cerebral cryptococcoma infiltration at the bilateral basal ganglia, so there was a possibility that the encephalitis by cryptococcus caused the increased CSFP.^2^ We speculate that this rapid exacerbation of the symptoms by markedly elevated CSFP is one of the causes of high mortality.

An increase in CSFP is associated with high mortality, so aggressive management for controlling CSFP is strongly recommended in the treatment guidelines of 2010 issued by the Infectious Diseases Society of America.^1^ In the current case, initial CSFP was very high, so that frequent lumbar punctures were required, and the signs of CSFP elevation developed suddenly. According to the guidelines,^1^ CSFP elevation should be managed with ventricular or continuous spinal drainage. Some reports have shown the efficacy and safety of continuous spinal drainage for severely raised CSFP^3^, ^4^ and this technique could be induced at the bedside, so we selected continuous drainage instead of ventricular. It was very effective for controlling markedly increased CSFP without complications (Fig. 1). However, CSFP in this case caused abrupt depressions in cerebral function, which might be very difficult to safely control with spinal drainage. So, a better option might be ventricular drainage that could control CSFP without a risk of brain herniation and be a direct monitor of intracranial pressure. Further studies are needed to define appropriate methods of drainage for controlling CSFP during severe meningitis.

In this case, BIS values dropped drastically before exacerbation of the symptoms by elevated CSFP. Furthermore, BIS values dramatically recovered during the lumbar puncture, suggesting that BIS monitoring could reflect changes in cerebral function due to alternations of the CSFP (Fig. 1). Bispectral index is a monitoring method for cortical electrical activity, and is often used to determine the depth of anesthesia.^5^ Some reports have suggested that BIS values are correlated with outcomes of patients with coma under the usage of a muscle relaxant.^6^, ^7^, ^8^ We could not measure absolute values of CSFP, so we could not assess changes in CSFP when BIS values dropped or the symptoms caused by elevated CSFP appeared. But the abrupt exacerbation of BIS values and the rapid recovery after CSF drainage indicated that further CSFP elevation was a cause of the sudden depression in cerebral function. Although BIS has several limitations in the interpretation of data, it could be a useful monitor for changing CSFP under anesthesia with neuromuscular blockade.

## Disclosure

Approval of Research Protocol: Not applicable.

Informed Consent: Informed consent was obtained from the patient for publication of this case report. A copy of the written consent is available for review by the Editor‐in‐Chief of this journal.

Conflict of Interest: None declared.
